# The Gastric Juice of the Horse

**Published:** 1888-07

**Authors:** R. Meade Smith


### EDITORIAL DEPARTMENT.

### THE GASTRIC JUICE OF THE HORSE.

BY R. MEADE SMITH, M.D.

In the horse the performance of gastric fistula is impossible on anatomical grounds, and various attempts have been made to collect gastric juice by performing œsophagotomy and passing a sponge through a tube into the stomach, or by killing the animal, tying the pylorus and collecting the contents. These methods, however, fail to secure a gastric secretion which is free from bile and pancreatic juice. So also the use of a stomach pump is rendered difficult or impossible on account of the long, pendulous soft palate and the discomfort and struggles which it causes in animals, when attempts to employ it are made. By means of the methods referred to above, the fluids obtained from the stomach will always contain saliva and various food products as well as true gastric secretion; but since these substances are always mixed in actual digestion, they will answer for the study of the process of digestion, even though they will prevent accurate statements as to the chemical constitution of this secretion.

The gastric juice in the horse is secreted only by the glands of the fundus, from a surface only about two hands' breadth in extent. Extracts from this portion of the mucous membrane contain more acid, more ferments, and what is remarkable, more mucine than extracts made from the pyoloric portion. Ellenberger and Hofmeister found that the degree of acidity immediately after eating was in the horse only 0.084 per cent. After an hour the acidity arose to 0.1 per cent. and still later to 0.2 per cent. Immediately after eating, no hydrochloric acid was present, but only appeared four or five hours after the commencement of the meal. Lactic acid was always present, apparently even in excess of hydrochloric. This may perhaps serve to explain the fact that in the horse's stomach the change of starch into sugar may go on even in the presence of 0.2 per cent. of acid, as organic acids interfere less than mineral acids with this process. The characteristics of the acids of the gastric juice have been found to depend upon the food. Thus, these authors have found that after feeding, the distribution of acids was as follows :

| | H.Cl. | Organic Acids. |
|---|---|---|
| 1. Oats and chopped straw, | 0.163% | 0.287% |
| 2. Oats, | 0.490% | 0.610% |
| 3. Hay, | 0.022% | 1.798% |

It is thus seen that when the food consists of oats the maximum percentage of H. Cl. is found in the gastric juice, while, when hay is given, the mineral acid falls to a minimum, while the organic acids are in excess. The importance of these facts is evident when it is remembered that oats contain 12 per cent. of albuminous matter and 65 per cent. of carbohydrates, including celulose, while hay contains only 9 per cent. of proteids, and 70 per cent. of carbohydrates. Hence, when oats are given the excess of hydrochloric acid is especially favorable for the peptonization of the proteid constituents, while it interferes with the digestion of carbohydrates. On the other hand, when hay is given, the excess of organic acid, as already mentioned, does not interfere with the action of ptyaline on starch, while still permitting the peptonization of the proteids.

The watery extract of the mucous membrane of the fundus differs, as already mentioned, from that of the pylorus. It contains more mucus, more acid and more ferment. The fundus extract contains a ferment which converts caseine, fibrine, albumen and gelatine into peptone in media, containing 0.15 to 0.05 per cent. of hydrochloric acid. While 0.6 per cent. H. Cl. arrests digestion, in the case of organic acids, it was found that the percentage might be considerably increased above this point and yet digestion go on. Thus, the gastric extract appeared to possess about the same degree of activity when lactic acid was present in two per cent. as with hydrochloric acid present in 0.2 per cent. The ferment is only diffusible with great difficulty and resists to a high degree putrefactive and alcoholic fermentation; lactic acid fermentation does not interfere with its activity.

The ferment is soluble in water, glycerine, dilute acids, alkaline and saline solutions, and loses its activity at sixty degrees centigrade. Such an artificial gastric juice, extracted from the mucous membrane of a horse's stomach, will digest animal tissues in the same way as extracts prepared from the mucous membrane of the stomach of a carnivora. Extracts prepared from inflamed mucous membrane are totally inactive. In addition to the pepsin, the gastric juice of the horse also contains the milk-curdling ferment and salts, lactic acid ferment, and traces of a diastatic ferment, all of which may be precipitated by alcohol. The distribution of these ferments and of the acidity of the gastric juice is the same as in the dog, with the exception that no secretion takes place in the membraneous cardiac portion of the organ.

Gastric digestion in solipedes is more important than one might be led to suppose, and continues, to a certain extent, from one meal to the next, as the residue remains in the stomach until the next one is taken, and, therefore, the stomach does not completely empty itself after twenty four hours. The character of this residue will, of course, vary with the nature of the food; thus, the contents of the stomach after feeding with oats, is a dry, crumbling mass, containing sixty to seventy per cent. of water. After feeding with hay, the percentage of water may be as much as eighty per cent.; the reaction is always acid.

Gastric juice obtained as above, by the method of Ellenburger and Hofmeister, rapidly converts starch into sugar, although the ferment is not derived from the stomach, but from the swallowed saliva.

The change of starch into sugar goes on in the stomach, as is proved by the fact that gastric juice, outside of the body, will turn starch into sugar, and by the fact that after feeding starch, large quantities of sugar may be found in the contents of the stomach. The digestion of starch is most active in the first two hours of digestion, and stops after five or six hours, when the percentage of hydrochloric acid is most marked.

---

SNAKE BITE AND ITS ANTIDOTE.—Dr. H. C. Yarrow, Curator Department of Reptiles, United States National Museum, has been publishing in the late numbers of the *Forest and Stream* an account of his experiments in discovering an antidote for the venom of serpents, and he believes such an antidote has been found in jaborandi, the South American plant *Pilocarpus pennatafolius.* In the articles an introductory review of former experiments and literature relating to serpent poison, from 1664, is given, also the account of some experiments made with certain remedies that have attracted the attention of herpetologists of late years, but with negative results. Among them he mentions permanganate of potash, arsenic, boneset, ammonia, alcohol, euphorbia, "snake stone," snake gall, etc.

As regards alcohol, which seems to be the generally used remedy nowadays, Dr. Weir Mitchell considers it as merely a "counteractive agent," a stimulus supply which may buoy the patient over the prostration produced by the venom, but as a direct antidote it fails, and this is proved by the fact that a mixture of alcohol and venom is no less deadly than the venom itself.

Jaborandi seems to be the favorite remedy at present, and two men, one a physician in Washington City, have so much confidence in it as an antidote that they have offered themselves for the purpose of experiments with the venom.

Mention is also made of the Gila monster, *Heloderma suspectum*, the bite of which was considered poisonous by many, especially by Drs. Weir Mitchell and Reinhert, who read a paper on the subject before the College of Physicians, of Philadelphia. The author, after many experiments with the venom, considers it harmless.

In conclusion, Dr. Yarrow gives the result of his experiments and study, and recommends the following treatment:

Bandage between parts bitten and heart. Scarification of wound, abstraction of poison by suction or cupping. Alcohol as a stimulant. Intoxication does no good. Hypodermic injection of 20 per cent. solution of permanganate of potash into the wounded tissue ; 20 minims fluid extract jaborandi or pilocarpine hypodermically until free perspiration and salivation is produced.

The writer does not endorse the preventive inoculation theory, but thinks there may be something in it.

In the Proceedings of the Canadian Institute, Vol. V. No. 2, is presented a paper by Dr. J. H. Garnier, in which he proposes iodide of potassium, say 1 drachm, followed in ten or fifteen minutes by sweet spirits of nitre, which resolves the iodide into its elements and sets the iodine free, which attacks and neutralizes the venom in the tissues.

C.

---

Epizootic of Glanders in Pennsylvania.—A recent investigation and examination of the horses of Monroe County, and adjacent territory in Pennsylvania, shows the existence of a widespread and dangerous epizootic of glanders farcy in that region. The history shows, that for several years, at least, the disease has been steadily on the increase. Under the enticing name of "distemper" and "influenza" animals, dropping ounces of yellowish viscous matter from their nostrils, and with submaxillary glands like a bunched mass of a rosary, have been treated by veterinarians! Imported veterinarians from New York State have confirmed the diagnosis and approved the treatment of their local colleagues. Market horses, from over the mountains in Northhampton County, have been driven in weekly, running at both nostrils profusely, their septa nasi riddled with ulcers, and their legs covered with farcy buttons and chancres, to be fastened to the posts of the market place, and to be baited in the mangers of the best hostelries.

The local practitioners have acted as agents for insurance companies and have insured these six months' cases of "distemper," and have paid for them, when they died of pneumonia.

A day's investigation showed a number of diseased animals well disseminated over the country, to make one believe that there must be hundreds of similar cases in the neighborhood. Other cases in the southern part of the Commonwealth are reported to be badly infected—Philadelphia County and city certainly has hundreds of cases of glanders and farcy on the streets, which should have (in the present course of events and procedure) been converted into toilet soaps long ago. It is especially dangerous in Philadelphia, as, from the class of stables which are infected, horses go constantly, to ease their badly shod feet, in the surrounding agricultural loam of New Jersey, Delaware, Maryland and Pennsylvania.

There is no specific law for glanders on the statutes of Pennsylvania. H.

---

Gallus Bankiva.—Through the kindness of Dr. R. W. Burke, A.V.D., of Jubbalpore, India, we have succeeded in procuring two specimens of the wild India fowl. A valuable paper on the osteology of this bird, by Dr. R. W. Shufeldt, U.S.A., will appear in the October number of the Journal. A series of papers on the myology of the *Gallus Bankiva* has been published in the proceedings of the London Zoölogical Society, and this we believe to be the only thing written on this species. C.

---

New Publications.—The Journal of Comparative Pathology and Therapeutics. W. & A. K. Johnston, Edinburgh and London.

This new venture in veterinary journalism promises, judging from the character of the first number, to be quite a success.

The editor is J. McFadyean, M.B., B.Sc., and he contributes an original and very interesting article on the Pathology of Hæmoglobinuria of Horses, finely illustrated, also one on Actinomycosis and Tuberculosis.

Professor Thomas Walley writes on Veterinary Sanitation, Dr. G. Sims Woodhead on the Morbid Anatomy and Histology of Pleuro-Pneumonia, Dr. Pottie on the

Propagation of Influenza from Stallions to Mares, and Dr. Campbell on the Surgical Treatment of Flatulent Colic in the Horse.

The editorial department has a good article on the Nature of Immunity and Protection in the case of Infectious Diseases, another on the Suppression of Pleuro-Pneumonia, etc., etc.

THE UNIVERSITY MAGAZINE is the title of a new monthly medical periodical published by A. L. Hummel, M.D., 224 South 16th street, Philadelphia, and the associate editors, of which are Drs. Pepper, Osler, Agnew, and other members of the staff of the University. The first number will appear on October 1st.

THE TEXAS HEALTH JOURNAL.—Our Texas brethren are progressive and are especially determined that the citizens of the "Lone Star" State shall be healthy and, consequently, wealthy. They are about to publish a monthly journal devoted to hygiene exclusively, and its motto will be "Public Health is Public Wealth." It will be edited by J. R. Briggs, M.D., Dallas, Texas.

MEDICAL CLASSICS.—The month of June brings us the first number of the second volume of the above-named periodical, the editor of which is Dr. Ferdinand Seeger. It is twice the size of former numbers and replete with exceedingly interesting reading matter, *e.g.*, "Summer Resorts," by John McMullen, A.M.; "How Best to Keep our Dogs in Health," by Dr. Gordon Stables, R.N.; "The Doctor in the Kitchen," by Dr. Seeger, which is quite a philosophical disquisition on gastronomy, and gives the best recipes for salads extant. "Summer Drinks," "Insect Pests," "Corpulency," etc., etc. C.

